# Recurrent Neural Model to Analyze the Effect of Physical Training and Treatment in Relation to Sports Injuries

**DOI:** 10.1155/2022/1359714

**Published:** 2022-09-30

**Authors:** Jyoti A. Dhanke, Rajesh Kumar Maurya, S. Navaneethan, Dinesh Mavaluru, Shibili Nuhmani, Nilamadhab Mishra, Ellappan Venugopal

**Affiliations:** ^1^Department of Science (Mathematics), Bharati Vidyapeeth's College of Engineering, Lavale, Pune 412115, India; ^2^Department of Computer Applications, ABES Engineering College, Ghaziabad 201009, Uttar Pradesh, India; ^3^Department of Electronics and Communication Engineering, Saveetha Engineering College, Chennai, Tamil Nadu, India; ^4^Department of Information Technology, College of Computing and Informatics, Saudi Electronic University, Riyadh, Saudi Arabia; ^5^Department of Physical Therapy, Imam Abdulrahman Bin Faisal University, Dammam 34212, Saudi Arabia; ^6^School of Computing Science and Engineering, VIT Bhopal University, Madhya Pradesh 466114, India; ^7^Department of Electronics and Communication Engineering, School of Electrical Engineering and Computing, Adama Science and Technology University, Adama, Ethiopia

## Abstract

Artificial intelligence has rapidly grown and has made the scenario that no field can function without it. Like every field, it also plays a vital role in the sports field nowadays. In certain sports, injuries happen very often due to heavy training and sudden speedy actions, especially in athletics and football. Here arises a need to analyze the effect of physical training in sportsperson by collecting data from their daily training. With the help of artificial intelligence, a recurrent neural model is developed to analyze the effect of physical training and treatment concerning sports injury. A Recurrent Neural Network (RNN) can be a subsection of Artificial Neural Networks (ANN) that uses the neural nodes connected in a temporal sequence. The temporal sequence is one of the essential terms in this research, which denotes a data sequence of events in a given timeframe. The recurrent neural model is an intelligent machine learning method that comprises a neural schema replicating humans. This neural schema studies the data it collects from the athletes/players and processes it by analyzing previous injuries. Sports injuries have to be analyzed because, in some cases, it becomes more dangerous to the sportsperson that they may even lose their career due to disability. Sometimes it may cause a massive loss to the club or company that hired the sportsperson for the sport. The prediction process can give the player rest until he recovers, thus becoming the safest approach in sports. Therefore, it is essential to analyze the sportsperson's track data to keep an eye on his health. In this research, RNN model is compared with the existing Support Vector Machine (SVM) in concerning to the effect of physical training and treatment for sports. The results show that the proposed model has achieved 99% accuracy, which is higher than the existing algorithm.

## 1. Introduction

A Recurrent Neural Network (RNN) model is a type of Artificial Neural Network (ANN) that aids in the system's dynamic behavior for a shorter duration. Additionally, the nodes in the networks are connected, which can construct a graph of directed or undirected categories over time. Athletes compete in a variety of events that require extreme fitness and stamina. Illness and aging produce anatomical and functional changes in the human body, putting senior people at risk of musculoskeletal and cardiovascular overload [1]. A substantial percentage of injuries (both acute and exceptional) are minor and can be treated with a bit of break from training and competition. The injuries of the sportsperson have to be treated as soon as possible and as effectively as possible [2]. However, it is important to note that inactivity and immobility have a more significant influence on structures and functions in the old than in the younger. Most physically active seniors are selected because they have better health and physical capacity than sedentary individuals their age, increasing their physical capacity. They will, however, be impacted by some of the drawbacks of physical overloading, mainly because the aging body's capacity to respond to high levels of loading is impaired [3]. Acute injuries are prevalent in senior persons who participate in ball games, downhill skiing, and gymnastics, which involve a lot of coordination, reaction time, and balance. Muscle has been described as the most usually acutely damaged tissue among active senior athletes. The most prevalent type of injury is to the lower extremities [4].

## 2. Literature Review

The RNN approach is designed to capture temporal correlations and is more successful for sequential data than traditional neural networks (NNs). After competitions, athletes frequently engage in rigorous physical conditioning and specific skill training. Regular training sessions for athletes are typical of a greater intensity. Athletes' physical and emotional health are harmed by long-term, high-intensity exercise, which leads to sports injuries. An injured athlete could not achieve the expected result in the competition due to his inability to undergo high-intensity training [5]. Based on the relevance of neural networks for target recognition, they present a novel dual-level feature fusion neural network (NN) model for sports injury estimate. The proposed model improves effective discrimination by building a dual-fusion structure with a 1 : 1 convolution and linkage to overcome the problem of feature loss. According to the trials, the suggested model exhibited a classification accuracy of 97.0 percent, a sensitivity of 95.70 percent, and a specificity of 97.54 percent [6]. The proposed model outperforms all of the other models that were taken into account for this research. Using a neural network to build a sports match prediction model and anticipate the outcomes can provide a theoretical framework for practice, prediction, and analysis. Various data mining techniques examine these medical records [7].

The Recurrent Neural Network (RNN) is a reliable technique that has been extensively used in machine learning and bioinformatics. The research on injury classification and level identification of the spinal cord is done using CT scan images that have been segmented using adaptive thresholding methods. The disc is then localized using the sparse fuzzy *C*-means clustering technique once the segments have been created. The next stage recovers the connectivity characteristics, statistical features, image-level features, grid-level features, Histogram of Oriented Gradients (HOG), and Linear Gradient Pattern (LGP) from the localized disc. After that, a Deep Convolution Neural Network based on the Crow Search Rider Optimization approach is used to identify damage (CS-ROA-based DCNN). Once the occurrence of the injury has been identified, the proposed Deep Recurrent Neural Network (Deep RNN) is used to categorize the injury severity, and the procedure is terminated if there is no damage [8]. The damage level is determined by the injury detection classifier, which might be normal, wedge, concavity, or crush [9].

A vast body of research has paved the way to improvements in legislation and policies to lessen the incidence and impact of concussions during the previous decade. Young individuals who engage in high-risk activities like sports, on the other hand, commonly underreport concussions, while others may exaggerate reports for a variety of reasons. Such laws and legislation must function within a supportive social context to be effective. Therefore, understanding the culture around concussion is crucial to decreasing concussion and its consequences [10]. An automated deep neural network approach is performed to analyze public opinion on concussion concerns in sports. As a result, one of the project's objectives is to develop a viable method for analyzing and measuring the public's current attitude toward sports-related concussions by examining an extensive collection of public views and perspectives on the subject. Various stakeholders may use this method's data to measure public opinion and attitudes around concussions. This knowledge might aid in the successful implementation of concussion-reduction strategies. Continuous monitoring of lab data and high-dimensional vital signs is needed to detect and diagnose acute patient issues, whereas continuous monitoring has been a difficult task in the past [11]. Recurrent Neural Networks (RNNs), a type of deep learning model, have lately demonstrated their capacity to predict such events. In actuality, however, patient data are continually being added, and RNN does not have a feasible adaptation mechanism to integrate this new data and enhance accuracy. The suggested method relies on the previous projected output and its associated label to update the RNN's hidden state. The additional information offered by the predicted past production and label improved the model's performance. This model's regularization technique considers the model's label prediction errors and its input data estimate errors [12]. The regularization approach reduces the variance of the model and the time gap of the self-correcting mechanism. The proposed model is suitable for both classification and regression. The proposed models were evaluated using real-world, large-scale ICU datasets. This method includes historical timestamp prediction mistakes into the current timestamp forecast, allowing the model to “learn” from previous forecasts. A regularization technique took into account the model's estimating errors on the input data and its label prediction errors [13]. As a result, an intelligent system such as Recurrent Neural Networks (RNNs) is required to efficiently analyze, predict, and recognize sports injuries.

## 3. Motivation of the Work

Physical education is recognized as an essential component of health education as well as an ability to reflect of a school system. A sports leader is in charge of students' physical education and/or health training but also treatment. Through student physical education classes at higher education institutions, this study suggested a technique for training but also treatment evaluation. The framework proposed by humans can improve the overall generalizability of the conventional BP network along with training time by integrating a neural network with the BP algorithm.

## 4. Proposed Model

**Figure 1:**
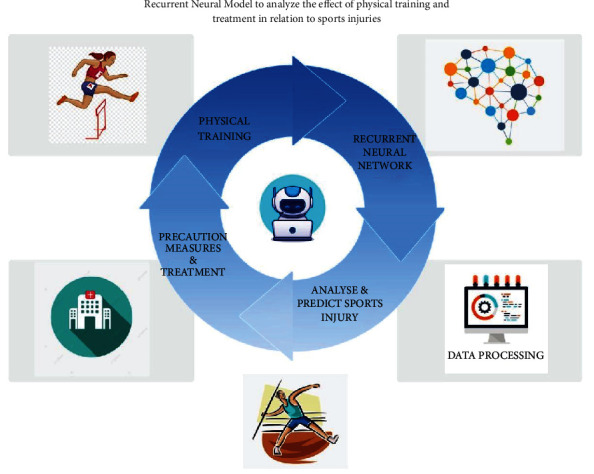
Proposed recurrent model for prediction and analysis of sports injuries.

Sport injuries can sometimes become more dangerous that it can cause disability to the sportsperson. This ends the career of the sportsperson and also causes big loss to the team who invested huge time and money on the particular player. Thus, a frequent monitor of their health is done periodically and the data are being recorded in a database. Each player/athlete has a separate kind of body health. In this scenario, one person's health data cannot be taken as a reference in treating other person beyond certain extent. Thus, the data of a player have to be compared and analyzed with his own dataset that was recorded previously. The data are collected and analyzed with the help of artificial neural networks. In this case, a Recurrent Neural Network (RNN) model has been used to analyze the effect of physical training and treatment in relation with sports injuries is depicted in the Figure 1.

The main phases involved in this method are as follows.

1. Physical Training: The physical training plays an important part in the career of a sportsperson. Without training session, the fitness level to play the game is lost even by star sportsmen. But over indulgence in training can cause physical stress to the players, leading to sports injuries like muscle tear. Therefore, training has to be done in a manner that the occurrence of sports injury has also to be avoided by predicting it beforehand and taking leave for the session or sport until the completion of the healing process.
2. Recurrent Neural Network: In order to predict the occurrence of a sports injury, an artificial neural network is assigned to accomplish the task. The ANN used here is Recurrent Neural Network (RNN), which analyzes the neural nodes with respect to the temporal sequence. The temporal sequence is a data collection method of collecting temporal data according to the sequence of happening in a given set of timeframe. These data are analyzed for predicting the sports injury.
3. Analyze and Predict Sports Injury: There is a strong need in predicting the occurrence of sports injury to avoid the loses that occur in one or more ways. In Recurrent Neural Network (RNN), the outputs of all the neurons are connected to the inputs of the neurons. The Recurrent Neural Network is used as a computational device system in analyzing the data to produce computational data output, which can be used as a predictive measure to avoid sports injuries.
4. Precaution Measures: With the help of the RNN output, the player is advised to take a break until complete healing process is done. This is also checked with the help of the medical support team and AI tools. Regarding AI tools used in the process, there are a number of AI sensors and biosensors that are already in use in the medical industry. Those biosensors are used to collect data in some cases. Thus, an intelligent RNN plays an important role in predicting the player's health to avoid sports injuries.

## 5. Proposed Work

The processing elements of such woman are *x*, which appear to be the analysis value of education physical quality education; the continental surface modules are *x*, and so are the extracted features *i* which appear to become the analysis significance of training physical quality education. Since the input image device transmits data directly to a frame node's center, the output of a input layer network equals a input; the information or data of a mid-layer access point seem to be the major contributors of an output node; and also the activation function alone has one network device, which also obtains the information of an intermediate layer access point but also produces an output its training but also treatment requirement evaluation effects.

Input data *m*_*i*_, *i*={1,2, . . . ., *x*}, where *x* reflects training and treatment quality evaluation.

The equation (1) is represented by the input to the *H* middle of the specimen node.

(1) $$\begin{array}{c} H_{j}=\overset{x}{\underset{j=1}{\sum }}\varphi_{ij}m_{i}\text{.} \end{array}$$

The result is as follows from the equation (2).

(2) $$\begin{array}{c} R_{j}=\overset{x}{\underset{j=1}{\sum }}\frac{1}{{\left\{1+{\left[{\left(\sum_{i=1}^{x}\varphi_{i}R_{j}\right)}^{-1}-1\right]}^{2}\right\}}^{\prime }}=\overset{x}{\underset{j=1}{\sum }}\frac{1}{\left[{\left(H_{j}^{-1}-1\right)}^{2}\right]}\text{.} \end{array}$$

In which *φ*_*j*_ signifies the resilience from an input datatype ground station *H*_*j*_^−1^ to a frame datatype center *j* *as* *well* *as* *R*_*j*_ signifies the data's factor, the *i*^*th*^ training as well as treatment quality evaluation index.

Node of input signal: There are only *S* nodes with in destination node (as shown in equation (3)), and also the information has been the high speed of the structure node's center point:

(3) $$\begin{array}{c} S=\overset{x}{\underset{j=1}{\sum }}\frac{1}{{\left\{1+{\left[{\left(\sum_{i=1}^{x}\varphi_{i}R_{j}\right)}^{-1}-1\right]}^{2}\right\}}^{2^{\prime }}}\text{.} \end{array}$$

The learning optimization technique is defined as even the mean score of such mean square sum ∑_*i*=1_^*x*^*φ*_*j*_*R*_*j*_ of such an error between such overall performances but also the measuring device value of *G* measurement techniques and the calculation is displayed in equation (4).

(4) $$\begin{array}{c} G=\left(\frac{1}{M}\right)\overset{m}{\underset{m=1}{\sum }}{\left[\underline{s}-s\right]}^{2}=\left(\frac{1}{M}\right)\overset{m}{\underset{m=1}{\sum }}G_{j}\text{.} \end{array}$$

Despite the BP neural network evaluation process's modifying *δG* framework, the goal of network training and treatment is to minimize *τ* by modifying the network's authentication and authorization. The training algorithm technique *δc*_*ij*_ is being used to modify its delinking as in the following equation (5).

(5) $$\begin{array}{c} G_{ij}=\sum \left\{\varphi_{ij}=-\tau \left(\frac{\delta G}{\delta c_{ij}}\right),\varphi_{j}=-\tau \left(\frac{\delta G}{\delta \varphi_{ij}}\right)\right.\text{.} \end{array}$$

Furthermore, for such a rate of learning, the *d*_*i*_*φ*_*j*_*R*_*j*_^2^ quantity of communication optimizing parameters here between the information data network and the medium layer wireless device is then shown in the equation (6).

(6) $$\begin{array}{c} \varphi_{ij}=d_{i}\varphi_{j}R_{j}^{2}\left[1-\overset{x}{\underset{i=1}{\sum }}\varphi_{ij}d_{j}\right]\omega_{j}\text{.} \end{array}$$

The number of connection optimizing parameters is as in the following equation (7).

(7) $$\begin{array}{c} \varphi_{i}=\overset{r}{\underset{j=1}{\sum }}S^{2}R_{j}\left[1-\overset{r}{\underset{j=1}{\sum }}\varphi_{j}R_{j}\right]{\left[\underline{s}-s\right]}^{2}\text{.} \end{array}$$

So, using this framework, its neural network's data transmission weight can also be described to use the ∑_*j*=1_^*r*^*φ*_*j*_*R*_*j*_ optimization technique of such a specific brain network and the imperfection between these overall performances, and also various sample values can be reduced. The framework will be in a higher optimization procedure, as shown in the equation (8), by using an optimized neural network.

(8) $$\begin{array}{c} \left(G\right)=\overset{r}{\underset{j=1}{\sum }}\int \left(\varphi_{1},\ldots \ldots \varphi_{n}\right)\text{.} \end{array}$$

For which (*G*) seems to be the overall lack of accuracy of network training and *φ*_1_, . . . . . , *φ*_*x*_ are the constant weights now since strong and secure numbering includes the weights of the network of input access to data points along with center layer access points and also the prediction models of concentrators and output node devices, and *n* represents the number of network weights. Among most of them, $\underline{s}$ and *s* are *φ*_1_ variables which it represent its upper and lower bounds of change.

The optimization algorithm is a low capacity problem in the process of analytical algorithms. Because an individual's areas of interest are roughly equivalent to ∫(*φ*_1_, . . . . . , *φ*_*x*_) ability, its representation of an optimization technique has a significant effect on genetic algorithms. Because of its close route planning friendship between both the *U* − *G* optimization procedure and the optimization techniques, the *G* < *U* strength training computational process described below equation (9) is being used.

(9) $$\begin{array}{c} \int_{i}=\underset{i=1}{\sum }\left\{\begin{array}{c} U-G\;G<U,0\;G\geq U \end{array}\right.\text{.} \end{array}$$

In which *e* represents the training optimization technique and *G* represents the sum of all *G* ≥ *U* in the current generation. The recommendations below are used by the *M*_*r*_ classification of genetic process parameters in order to accept responsibility for such $\underset{1}{\int }\left(U-G\right)/U\in \left[0,0.6\right]$ efficiency of integration and also to eliminate excess integration caused by important evolutionary declassification.

(10) $$\begin{array}{c} M_{r}=\left\{2\left(\frac{\int_{1}\begin{array}{cc} U-G & G<U \end{array}}{U}\right)\right\},\frac{\int_{1}\left(U-G\right)}{U}\in \left[0,0.5\right],\left[1-2\left(1-{\left(\frac{\int_{1}\begin{array}{cc} U-G & G<U \end{array}}{U}\right)}^{2}\right)\right],\frac{\int_{1}\left(U-G\right)}{U}\in \left[0.5,1\right]\text{.} \end{array}$$

The min  *an* *d* max technique is used for normalization handling ∫_1_(*U* − *G*)/*U* ∈ [0.5,1] since it is a successful implementation for processing information that can successfully retain its very own original definition while having caused no redundant data. The normalization equation (10) is to use this report for such input data.

The procedures of compacting a large variety of information into the field of view [0, 1] are known as normalization ∫_1_(*U* − *G*)/*U* ∈ [0.5,1]. The solution to the normalization is given in the equation (11) and is as follows.

(11) $$\begin{array}{c} d^{\prime }=\overset{x}{\underset{i=1}{\sum }}\frac{d-d_{\min}}{d_{\max}-d_{\min}}\text{.} \end{array}$$

The standardization process *σ* entails converting the dataset's small and big outlier documentation into a discrete random variable with such an average overall value of 0 and then a confidence level of 1. The following equation (12) depicts the standardization process.

(12) $$\begin{array}{c} d^{\prime }=\overset{x}{\underset{i=1}{\sum }}\frac{d-{\underline{d}}_{\min}}{\sigma }\text{.} \end{array}$$

Each data center is composed of three layers: input nodes, hidden nodes, and also convolution layers *β*, *γ*, *and* *α*, also with weight training of each surface being and also including. It suggests that specific *φ*_*y*′*y*_*q*_*y*′_^*t*−1^ information is managed to retain in RNN receptors after each cycle of data transmission. It must enter a *φ*_*y*_(*q*_*y*′_^*t*−1^) next nerve cell as new information and effect the subsequent data output. The equation (13) for such corresponding input nodes, its original input of both the hidden units, and also the outcome variable to it of destination node at time step *t* is represented in the following: equations (14) and (15).

(13) $$\begin{array}{c} \beta_{y}^{t}=\overset{M}{\underset{i=1}{\sum }}\varphi_{iy}d_{i}^{t}+\overset{M}{\underset{y^{\prime }}{\sum }}\varphi_{y^{\prime }y}q_{y^{\prime }}^{t-1}\text{,} \end{array}$$

(14) $$\begin{array}{c} \alpha_{y^{\prime }}^{t-1}=\varphi_{y}\left(q_{y^{\prime }}^{t-1}\right)\text{,} \end{array}$$

(15) $$\begin{array}{c} \gamma_{y^{\prime }}^{t-1}=\overset{M}{\underset{y=1}{\sum }}\varphi_{y0}q_{y}^{t}\text{.} \end{array}$$

As a result, by modeling differently in time-distance, this framework is enhanced much further, seeking to make this more distinctive than spatial transformation. The distributed probability, which gets to know to extract temporal features from streaming video using learning algorithm, is being used in the case as part of a hierarchy approach. As the introducing module, its own interpolation constricted machine effort in learning the hierarchy organization of the original data structure. The framework has become more difficult since it develops from highest to lowest level. The geographically but also temporally High Duplicate Network is named after a constant growth in interpretations.

Previously, the rapacious hierarchical model was used to train the Enlarged Deep Learning (Deep Belief Network (DBN)) model. Furthermore, each framework's input layers are informed at random, beginning with the lowest layer. The probability recognition of the hidden nodes is then reorganized, and understanding to some other layer is gained. This process was repeated indefinitely during training until all of the layers have been trained, since training the entire network might recover the hidden and exposed possibility representations of every particular part within the video.

## 6. Results and Discussion

**Figure 2:**
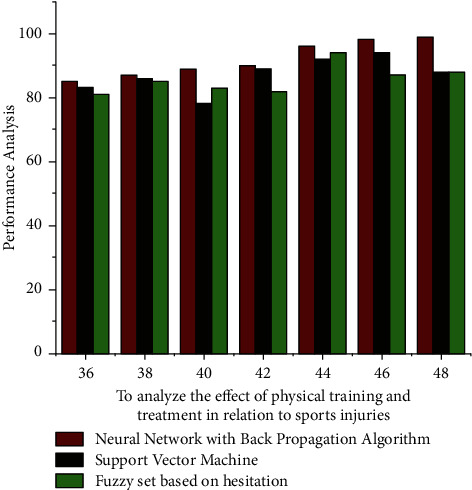
The computation accuracy of measurement weight results is compared.

The above graph having to learn optimization method is defined as the average score of these mean square ∑_*i*=1_^*x*^*φ*_*j*_*R*_*j*_ of such a mistake between such overall performance and the measuring handset value of *G* measurement methods. As in equation (4), Figure 2 depicts the physical education training and treatment performance of fourth- and eighth-grade students. The effectiveness of training and treatment is evaluated using a Recurrent Neural Network Model, a Support Vector Machine, and a Fuzzy Set Model based on hesitation. The computation is carried out by combining the performance of the fourth- and eighth-grade students. The proposed algorithm, Recurrent Neural Network Model, has a lower percentile at the early stage of new technology. However, at a later stage, the algorithm was able to produce results that were comparable to the Support Vector Machine. The fuzzy model yields results that are indistinguishable from the Recurrent Neural Network Model and Support Vector Machine (SVM) algorithms. Such a comparative graph demonstrates how repeated training, treatment, and learning can improve the performance of students and teachers when using the Recurrent Neural Network Model.  Table 1 shows that its Recurrent Neural Network Model provided small training accuracy than other algorithms for an average evaluation weight of 38. Furthermore, the accuracy rate of the Recurrent Neural Network Model is steadily improving compared to the other two algorithms with fluctuating results. Later on, the proposed algorithm achieved an accuracy of 99%, which is a minimum increase of nine percentage points over the Support Vector Machine and then a 18% increase over the Fuzzy set model.

**Figure 3:**
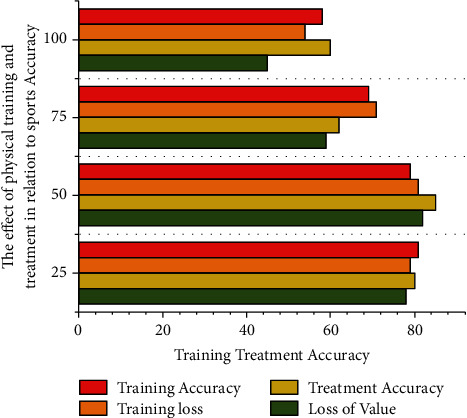
The effect of physical training and treatment on neural network accuracy but also loss in sports using the BP algorithm.

The *δG* framework is being modified as part of the BP neural network evaluation process. The goal of capacity training, as well as treatment, seems to be to minimize *τ* by modifying Internet backbone authentication process. To adjust its delinking, the developer these skills technique *δc*_*ij*_ is used resemblance of performance measures with those of algorithms represent in Figure 3 also demonstrates that such built model performance when compared conventional algorithms, including such neural network models, through seeking to resolve activity recognition problems. The 98.55% training and treatment accuracy rate also confirms its high specificity. The convolutional neural network could capture the qualities of training (93.26%), training and treatment (95.47%), accuracy and loss of training (88.74%) analysis, optimize the required data, and improve the consistency of exercise physiology recognition simply by attempting to adjust the set of parameters. The neural network accuracy training and testing graph is capacity forecast in physical education training and treatment based on BP neural network.

**Figure 4:**
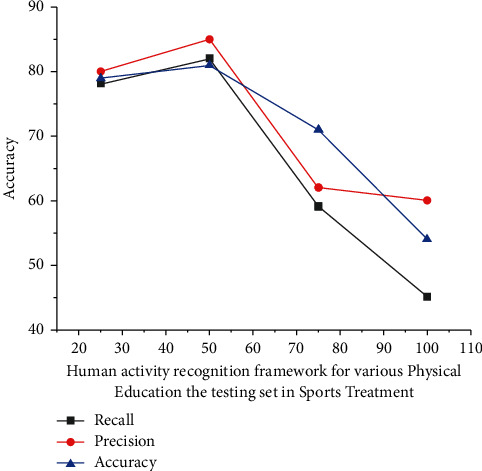
Performance evaluation of human activity recognition.

Moreover, with such a learning algorithm, the *d*_*i*_*φ*_*j*_*R*_*j*_^2^ quantity of communication optimization parameters between both the information data connection and the medium surface wireless device is shown in equation (6) elsewhere here represented in Figure 4; it also shows that trained human activity recognition framework appears to have an evaluation of overall accuracy of much more than 99.7%, precision of much more than 92.46 percent, recall frequency of many more than 89.72%, for the different topics; until training, the activity recognition framework for several Physical Education.

The isolation of visual medium, which includes higher rates of physical and also postures judgment in physical training and treatment, has been shown to continue providing educators with unbiased information about student movements, and thus, this recognition of educational body actions may provide legitimate input to improve training performance. Information from intelligent smart wearable sensors has been used to accurately determine human behavior, but the classification of constructions is a controversial subject. The experimental results (refer to Table 2) show that such a convolutional network-based activity recognition system could indeed accurately recognize human behavior with an accuracy of more than 98%. As a result of the Analysis for Multiple Physical Education assessment set in diverse physical education for precision (89%), recall (87%), and accuracy (99%), as well as the Human Sports Activity of precision (90%), recall (85%), and accuracy (98%).

**Figure 5:**
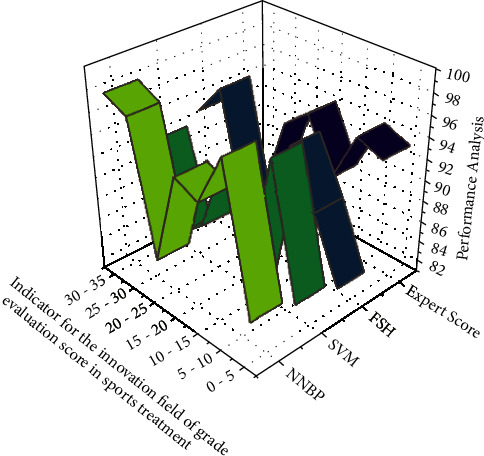
Analysis of evaluation score comparison results in sport training and treatment in physical education.

The suggestions below are being used by the *M r* categorization of genetic process variables to take accountability for such ∫_1_(*U* − *G*)/*U* ∈ [0,0.6] integration efficiency and to remove unnecessary integration caused by significant evolutionary release of information. The evaluation process of physical education courses follows the training, treatment, and learning processes and the analysis is shown in Figure 5. When compared to expert evaluation, the proposed automatic evaluation via the Recurrent Neural Network model outperformed many assessments. The graph's peaks represent the evaluation value for the specific assessment for students in both grades.  Table 3 shows the numerical representation. According to this table, the proposed Recurrent Neural Network model demonstrated fluctuating results during the learning process; however, it demonstrated consistent and increased performance at a later stage. Towards the final considered evaluation weight range of 30–35, the proposed system outperformed the existing Support Vector Machine, fuzzy set predicated on doubt, but also expert score by 6%, 5%, and 16%, respectively.

**Figure 6:**
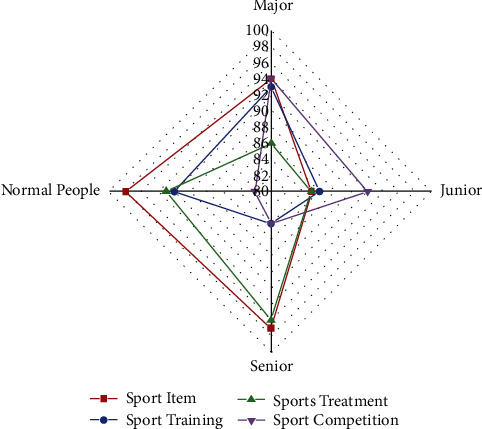
Analysis for result in the physical training and treatment.

Figure 6 depicts the analysis of results performed on students of various grades again for physical education training, treatment, but also learning process. Students are encouraged to practice on a regular basis in order to be eligible for competitions in any sport. The graph above shows that the analysis is performed on people from four different categories, including normal people, majors, juniors, and seniors, using sports items, competition, and practice as parameters. All of the necessary equipment for practicing and participating in any specific sport is included in a sports item. In this study, it is assumed that the player has all of the necessary resources. Among some of the four categories of people, the average person with no prior experience was only able to achieve 16% in the competitions despite having access to all of the necessary resources. Individuals in the major, junior, but also senior categories, on the other hand, perform better by 23%, 35%, and 43%, respectively. Every data center is made up of three layers: input endpoints, hidden nodes, and convolution layers *β*, *γ*, *and* *α* with weight training of every surface included. It implies that specific *φ*_*y*′*y*_*q*_*y*′_^*t*−1^ information is managed to maintain in RNN receptor sites after each data transmission cycle. It must enter the next neuron as new information and affect the subsequent data output.

**Figure 7:**
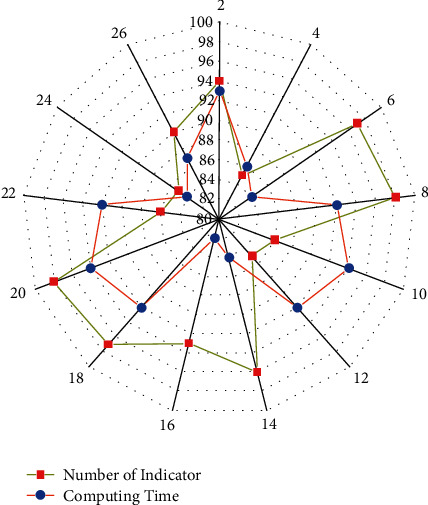
The thorough outcomes of sports training and treatment are critical.

The data in Figure 7 above demonstrate that training is required to participate in any sport, and learning them from a coach is also very important. The number of indicator or exercises increases with changes in this graph, as does the computing time or the players' duration of achievement. Initially, each person will devote the maximum amount of time to simple and short exercises. The duration of computation will be reduced as the players perform workouts and practices on a regular basis. It must enter the next neurone as new information as well as affect the subsequent data output. Figure 7 depicts the equations (13), for such corresponding input nodes, its original input of both hidden units, and the outcome variable to it of the destination node at time step *t*.

## 7. Conclusion

Injuries are most common among the sports persons during practice sessions and event participation. It is mandatory for them to recover from the injuries at the earliest and start a new beginning. In the technological development, physical training and treatment for the sports injuries can be monitored with the implementation of the intelligent system. This advancement in the technology aids the sports person and the coach to make regular updations on the status of improvement on the injuries concerning to the practice and treatment. In this research, Recurrent Neural Model is designed to perform frequent monitoring of the processes involved in sports injuries and predict the possibilities of injuries. The proposed model outperformed the existing Support Vector Machine, fuzzy set predicated on doubt, but also expert score by 6%, 5%, and 16%, respectively.

## Figures and Tables

**Table 1:** Analyze the effect of physical training and treatment on sports injuries using a result analysis. Precision in computing.

Effect of physical training and treatment in relation to sports	Recurrent Neural Network model (%)	Support Vector Machine (%)	Fuzzy set based on hesitation (%)
36	85	83	81
38	87	86	85
40	89	78	83
42	90	89	82
44	96	92	94
46	98	94	87
48	99	88	88

**Table 2:** Analysis for the result indifferent physical education the training set in sport treatment.

	Precision (%)	Recall (%)	Accuracy (%)
Various physical education	0.88	0.89	0.99
Human sports activity	0.91	0.86	0.97

**Table 3:** Comparison result analysis for different algorithm in the effect of physical training and treatment in relation to sports injuries.

Effect of physical training and treatment	Recurrent Neural Network model (%)	Support Vector Machine (%)	Fuzzy set based on hesitation (%)	Expert score
0–5	85	84	83	93
5–10	98	96	89	94
10–15	93	83	94	89
15–20	94	93	86	94
20–25	85	86	85	92
25–30	97	84	96	84
30–35	98	92	93	82

## Data Availability

The data used to support the findings of the study can be obtained from the corresponding author upon request.

## References

[1] Meng L., Qiao E. (2021). Analysis and design of dual-feature fusion neural network for sports injury estimation model. *Neural Computing & Applications*.

[2] Chantamit-o-pas P., Goyal M. Long short-term memory recurrent neural network for stroke prediction.

[3] Tirdad K., Dela Cruz A., Sadeghian A., Cusimano M. (2021). A deep neural network approach for sentiment analysis of medically related texts: an analysis of tweets related to concussions in sports. *Brain Inf*.

[4] Du H., Pan Z., Ngiam K. Y., Wang F., Shum P., Feng M. (2021). Self-correcting recurrent neural network for acute Kidney injury prediction in critical Care. *Health Data Science*.

[5] Kallinen M., Markku A. (1995). Aging, physical activity and sports injuries. *Sports Medicine*.

[6] Wang X., Jiang C. (2021). Computer-aided physical training sports injury risk simulation based on embedded image system. *Microprocessors and Microsystems*.

[7] Zadeh A., Taylor D., Bertsos M., Tillman T., Nosoudi N., Bruce S. (2021). Predicting sports injuries with wearable technology and data analysis. *Information Systems Frontiers*.

[8] Dobrosielski D. A., Sweeney L., Lisman P. J. (2021). The association between poor Sleep and the incidence of sport and physical training-related injuries in Adult athletic Populations: a Systematic Review. *Sports Medicine*.

[9] Kozin S., Kozina Z., Korobeinik V. (2021). Neuro-muscular training for injury prevention of students-rock climbers studying in the specialty “Physical Education and Sports”: a randomized study. *Journal of Physical Education & Sport*.

[10] Xianguo S., Cong W. Research on the Application of artificial intelligence technology in physical training.

[11] Li D., Yi C., Gu Y. (2021). Research on College physical education and sports training based on Virtual Reality technology. *Mathematical Problems in Engineering*.

[12] Coleman N., Coleman N. (2021). Pediatric athlete development and Appropriate sports training. *Common Pediatric Knee Injuries*.

[13] Balberova O. V. (2021). Candidate genes and single-nucleotide gene variants associated with muscle and tendon injuries in cyclic sports athletes. *Personalized Psychiatry and Neurology*.

